# Antibiotic treatment modulates protein components of cytotoxic outer membrane vesicles of multidrug-resistant clinical strain, *Acinetobacter baumannii* DU202

**DOI:** 10.1186/s12014-018-9204-2

**Published:** 2018-08-31

**Authors:** Sung Ho Yun, Edmond Changkyun Park, Sang-Yeop Lee, Hayoung Lee, Chi-Won Choi, Yoon-Sun Yi, Hyun-Joo Ro, Je Chul Lee, Sangmi Jun, Hye-Yeon Kim, Gun-Hwa Kim, Seung Il Kim

**Affiliations:** 1 Drug and Disease Target Team, Korea Basic Science Institute, Cheongju, 28119 Republic of Korea; 2 Center for Convergent Research of Emerging Virus Infection, Korea Research Institute of Chemical Technology, Daejeon, 34114 Republic of Korea; 3 Bio-Analysis Science, University of Science and Technology, Daejeon, 34113 Republic of Korea; 4 KBNP Technology Institute, KBNP, INC., Anyang, 14059 Republic of Korea; 5 Department of Microbiology, Kyungpook National University School of Medicine, Daegu, 41944 Republic of Korea; 6 Protein Structure Team, Korea Basic Science Institute, Cheongju, 28119 Republic of Korea

**Keywords:** Proteomics, *Acinetobacter baumannii*, Outer membrane vesicles, Modulation by antibiotic treatment

## Abstract

**Background:**

Outer membrane vesicles (OMVs) of *Acinetobacter baumannii* are cytotoxic and elicit a potent innate immune response. OMVs were first identified in *A. baumannii* DU202, an extensively drug-resistant clinical strain. Herein, we investigated protein components of *A. baumannii* DU202 OMVs following antibiotic treatment by proteogenomic analysis.

**Methods:**

Purified OMVs from *A. baumannii* DU202 grown in different antibiotic culture conditions were screened for pathogenic and immunogenic effects, and subjected to quantitative proteomic analysis by one-dimensional electrophoresis and liquid chromatography combined with tandem mass spectrometry (1DE-LC-MS/MS). Protein components modulated by imipenem were identified and discussed.

**Results:**

OMV secretion was increased > twofold following imipenem treatment, and cytotoxicity toward A549 human lung carcinoma cells was elevated. A total of 277 proteins were identified as components of OMVs by imipenem treatment, among which β-lactamase OXA-23, various proteases, outer membrane proteins, β-barrel assembly machine proteins, peptidyl-prolyl cis–trans isomerases and inherent prophage head subunit proteins were significantly upregulated.

**Conclusion:**

In vitro stress such as antibiotic treatment can modulate proteome components in *A. baumannii* OMVs and thereby influence pathogenicity.

**Electronic supplementary material:**

The online version of this article (10.1186/s12014-018-9204-2) contains supplementary material, which is available to authorized users.

## Introduction

*Acinetobacter baumannii* is a major Gram-negative bacterial pathogen that causes nosocomial infections such as ventilator-associated pneumonia, bacteraemia and urinary tract infections [1]. Like most Gram-negative bacteria, *A. baumannii* secretes outer membrane vesicles (OMVs), as first demonstrated using the *A. baumannii* DU202 multidrug-resistant (MDR) clinical strain that is cytotoxic and elicits a potent innate immune response in the host [2–4]. Various peculiar biological functions of *A. baumannii* OMVs have been elucidated. Vaccination of whole *A. baumannii* OMVs alone or in combination with biofilm-associated protein (Bap) effectively protects against *A. baumannii* infection and elevates innate immunity [5–7]. Furthermore, the plasmid-borne *bla*_oxa-24_ gene has been transferred into the carbapenem-susceptible *A. baumannii* ATCC 17978 strain using carbapenem-resistant *A. baumannii* OMVs as a vehicle for horizontal gene transfer [8]. Therefore, elucidation of the biological roles of the protein components of OMVs is important for understanding their relevance to pathogenicity.

Numerous physiological and environmental factors are known to influence OMV secretion in Gram-negative bacteria. For example, OMV secretion is much more pronounced in enterotoxigenic *Escherichia coli* than nonpathogenic wild-type or mutant strains [9, 10]. Additionally, antibiotics such as gentamicin, polymyxin, d-cycloserine and mitomycin C increase secretion of OMVs from *Pseudomonas aeruginosa* and *Shigella dysenteriae* [11–13], and high temperature, oxidizing agents and nutrients also act as stimulatory factors for OMV production [14].

In the *A. baumannii* DU202 MDR clinical strain, proteomic variation in the membrane-associated protein fraction, especially among outer membrane proteins and transporters, has been correlated with antibiotic stress following treatment with imipenem and tetracycline [15]. This indicates that proteomic variation in OMVs produced by *A. baumannii* DU202 may occur under specific antibiotic conditions.

In the present study, we found that the production of *A. baumannii* DU202 OMV was increased by imipenem treatment, and became more cytotoxic toward cultured host cells. We recently reported the complete genome of *A. baumannii* DU202 [16], and here we used this resource to perform proteogenomic analysis of protein components of OMVs following antibiotic treatment. Bacterial OMVs play important role as potent bacterial virulence factors [17] and a high incidence of resistance to imipenem has been reported for clinical *A. baumannii* strains in hospitals [18, 19]. This suggests that OMVs produced under imipenem treatment might be crucial to infection; hence their characterization may be clinically important.

## Methods

### Bacterial strain and growth conditions

*Acinetobacter baumannii* DU202 cells were cultured in Luria-Bertani (LB) broth to late exponential phase (optical density of 1.0 at 600 nm) for OMV preparation. LB broth was supplemented with imipenem or tetracycline (50 µg/ml) as required.

### Isolation and purification of *A. baumannii* OMVs

OMVs of *A. baumannii* DU202 were purified from bacterial culture supernatants as described previously [2]. Briefly, bacterial cells were removed by centrifugation at 6000×*g* for 30 min and supernatants were filtered through a 0.2 µm vacuum filter to remove residual cells and cellular debris. OMVs were ultra-filtrated and concentrated using a QuixStand Benchtop System (GE Healthcare, USA) with a 500 kDa hollow fibre membrane (GE Healthcare). Collected OMVs were precipitated by ultracentrifugation at 150,000×*g* for 3 h at 4 °C, and pellets containing OMVs were suspended in 0.5–1.0 ml of phosphate-buffered saline (PBS). OMV solution was further purified by sucrose gradient centrifugation (2.5, 1.6 and 0.6 M sucrose) at 200,000×*g* for 20 h at 4 °C. Sucrose was removed from each layer by ultracentrifugation at 150,000×*g* for 3 h at 4 °C, and purified OMVs were used for sterility tests and stored at − 80 °C until needed.

### Transmission electron microscopy (TEM)

Transmission electron microscopy (TEM) of OMVs was performed as described previously [20]. Briefly, OMV fractions were diluted with PBS, centrifuged at 150,000×*g* for 3 h, resuspended in PBS, applied to 400-mesh copper grids, stained with 2% uranyl acetate and visualized on a TEM instrument (FEI, USA) operating at 120 kV.

### Sodium dodecyl sulphate–polyacrylamide gel electrophoresis (SDS–PAGE) and in-gel digestion

Sodium dodecyl sulphate–polyacrylamide gel electrophoresis (SDS–PAGE) and in-gel digestion were performed as previously described [21]. The protein concentration of purified OMVs was determined using a modified BCA assay kit (Thermo Fisher Scientific). Protein components of OMVs (15 µg) were separated by 12% SDS–PAGE and divided into eight fractions according to molecular weight. Sliced gels were destained in destaining solution (10 mM ammonium bicarbonate and 50% acetonitrile). After drying, gels were incubated with reducing solution (10 mM dithiothreitol and 100 mM ammonium bicarbonate) at 56 °C, and iodoacetamide (55 mM) was added to alkylate cysteine residues of disulphides. Gels were washed in 2–3 volumes of distilled water and dried in a speed vacuum concentrator. After immersing dried gels in 100 µl of 50 mM ammonium bicarbonate, 7–8 µl of trypsin solution (0.1 µg/µl) was added and samples were incubated at 37 °C for 12–16 h. After tryptic digestion, samples were transferred into a new tube and 50 mM ammonium bicarbonate followed by 50% acetonitrile containing 5% trifluoroacetic acid (TFA) was added to recover tryptic peptide mixtures. The resulting peptide extracts were pooled and lyophilised.

### Proteome analysis by liquid chromatography combined with tandem mass spectrometry (LC-MS/MS)

Tryptic peptide mixtures were dissolved in sample buffer (0.1% formic acid and 0.02% acetic acid) and loaded onto a 2G-V/V trap column (Waters, USA). Concentrated peptides were directed onto a 10 cm × 75 μm (i.d.) C18 reversed-phase column at a flow rate of 300 nl/min. HPLC conditions and search parameters for tandem mass spectrometry (MS/MS) analysis were applied as described previously [20]. All MS and MS/MS spectra obtained using the LTQ-Velos ESI ion trap mass spectrometer were acquired in data-dependent mode (Thermo Fisher Scientific, USA). For protein identification, nano liquid chromatography (LC)-MS/MS spectra were searched using MASCOT version 2.4 (Matrix Science, UK) using protein sequences from the genome of *A. baumannii* DU202. The exponentially modified protein abundance index (emPAI) was generated using MASCOT (Matrix Science) [22]. MS/MS analysis of each sample was performed at least in triplicate.

### Analysis of OMV production following treatment with stressor molecules

Treatment with stressor molecules was performed s described previously [11, 23]. Briefly, pre-cultures of *A. baumannii* DU202 were inoculated into 250 ml of LB broth and grown to mid-log phase (OD_600_ ~ 0.5) at 30 °C with vigorous shaking (180 rpm). Cells were harvested by centrifugation at 6000×*g* for 30 min and resuspended in 250 ml of fresh LB medium at 30 °C. Hydrogen peroxide, d-cycloserine and polymyxin B were added separately as required at final concentrations of 1 mM, 250 µg/ml and 2 µg/ml, respectively. To analyse the effect of hydrogen peroxide, fresh reagent was added to the culture every hour and OD_600_ measurements were taken. *A. baumannii* DU202 cells cultured in LB broth alone served as a negative control.

### Animal cell culture and apoptosis assay

A549 human lung carcinoma cells were cultured in RPMI 1640 culture medium supplemented with heat-inactivated 10% foetal bovine serum (FBS) under humidified 5% CO_2_ and 95% air at 37 °C. Cells were plated onto 12-well culture plates, and OMVs were applied and incubated for 24 h. For apoptosis assays, cells were stained with fluorescein isothiocyanate (FITC)-conjugated annexin V, propidium iodide (PI) and Hoechst reagent according to the manufacturer’s instructions. Stained cells were analysed using a NucleoCounter NC-3000 image cytometer (ChemoMetec, Denmark) [20].

### Bioinformatic analysis

The subcellular locations of proteins were predicted using the subcellular location prediction program PSORTdb 2.0 (http://db.psort.org/). Transmembrane helices in membrane proteins were predicted using the TMHMM server version 2.0 (http://www.cbs.dtu.dk/services/TMHMM-2.0/). The phage region in genome of *A. baumannii* DU202 was analysed with PHAST [24]. Spearman correlation coefficient and scatter plots between each sample were calculated by R language (http://www.r-project.org) using value of protein abundance according MASCOT results.

### Western blotting and immunoproteomics analysis

Rabbit OMV_DU202_ antiserum was prepared with technical assistance from Young In Frontier, Inc. (Seoul, Korea). Three injections were applied at intervals of 2 weeks, and blood was collected 1 week after the final injection. At 2 weeks after the third injection, serum was obtained by retro-orbital bleeding. For western blotting, OMV protein samples were separated by 12% SDS–PAGE, protein bands were transferred to a nitrocellulose membrane (Bio-Rad, CA) and the membrane was washed with TRIS-buffered saline (TBS) after blocking with 5% skim milk in TBS for 1 h. Following incubation with antiserum (1:4000 in 3% skim milk in TBS) for 14 h at 4 °C, the membrane was washed with TBST (0.5% Tween 20 in TBS) and specific IgG binding was visualised by incubation with anti-rabbit-IgG peroxidase conjugate (1:4000 in 3% skim milk in TBS) and development with a chemiluminescent substrate (GE Healthcare). The chemiluminescence signal was detected using an ImageQuant LAS 400 mini (GE Healthcare). A separate gel was used for protein identification by LC-MS/MS analysis.

## Results and discussion

### Antibiotics and stressor molecules induce differential production of OMVs in *A. baumannii*

**Fig. 1:**
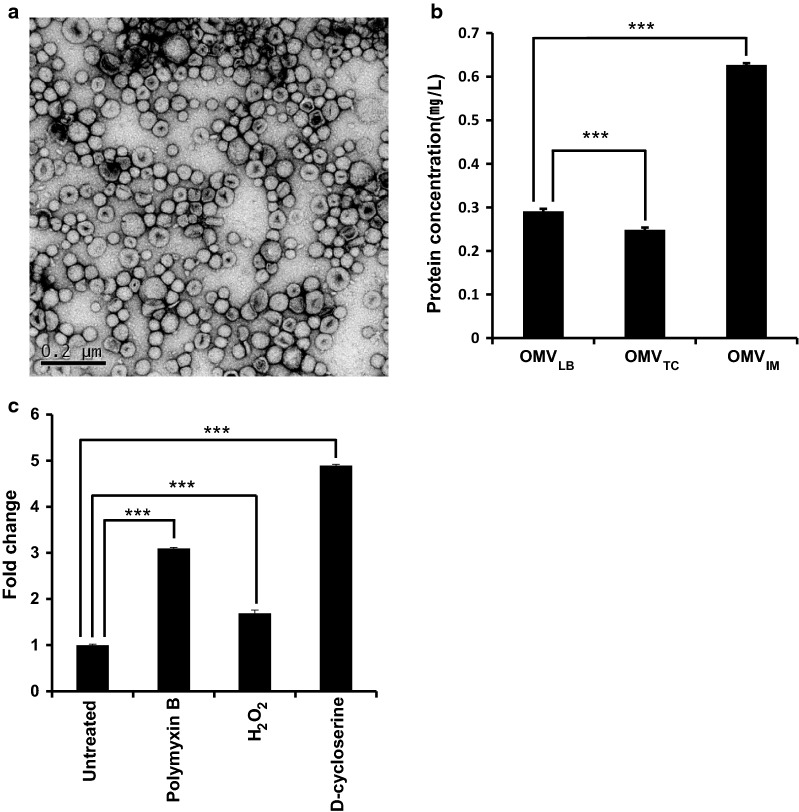
Differential production of *A. baumannii* DU202 OMVs according to antibiotics and stressors. Transmission electron micrograph of OMVs prepared from LB medium supplemented with imipenem (OMV_IM_) (**a**). Differential production of OMVs following treatment with antibiotics (**b**) and stressor molecules (**c**). Data are shown as means ± SD. ***p value < 0.001

OMVs of *A. baumannii* DU202 were purified and designated as OMV_LB_ (OMVs from LB culture condition), OMV_IM_ (OMVs from imipenem culture condition) and OMV_TC_ (OMVs from tetracycline culture condition) according to the culture conditions. Electron microscopy (EM) analysis revealed that purified OMVs were homogeneous (Fig. 1a), but the overall amount produced varied with the culture conditions (Fig. 1b and c). OMVs were increased > 2.2-fold following exposure to imipenem compared with untreated controls (Fig. 1b). Imipenem is an inhibitor of β-lactamases that inhibits cell wall synthesis in Gram-positive and Gram-negative bacteria [25]. Stressor molecules d-cycloserine, polymyxin and hydrogen peroxide were also tested, and d-cycloserine caused the largest increase in OMV production (Fig. 1c). d-cycloserine is a peptidoglycan inhibitor [11, 26], which indicates that weakening the integrity of the *A. baumannii* cell wall stimulates OMV production. By contrast, tetracycline, a protein synthesis inhibitor targeting the ribosome, had no effect on OMV production (Fig. 1b).

### Pathogenicity of *A. baumannii* OMVs against cultured epithelial cells

**Fig. 2:**
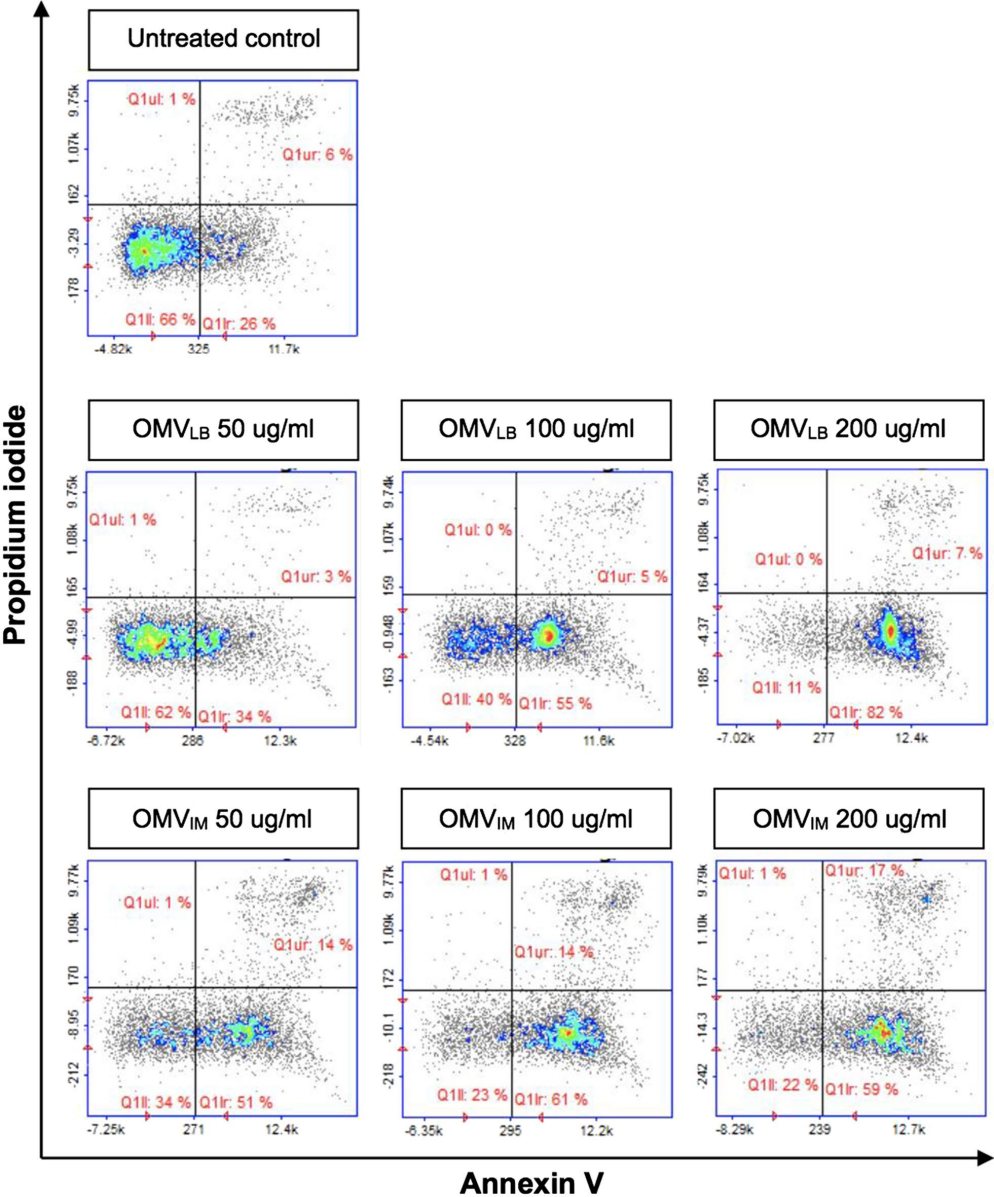
Cytotoxic effect of *A. baumannii* DU202 OMVs. A549 human lung carcinoma cells were treated with various concentrations (0, 50, 100 and 200 µg/ml) of OMVs for 24 h, and apoptosis was assessed

*Acinetobacter baumannii* OMVs are known to be cytotoxic toward animal host cells [3]. To investigate the cytotoxicity of *A. baumannii* DU202 OMVs, A549 human lung carcinoma cells were treated with different concentrations of OMV_LB_ or OMV_IM_. OMV_LB_ showed moderate early apoptosis-stimulating activity, whereas OMV_IM_ induced severe apoptotic cell death at the same concentration (Fig. 2). OMV_TC_ also exhibited cytotoxicity toward host cells (data not shown). These results indicate that OMVs isolated from *A. baumannii* treated with antibiotics are more cytotoxic, and this prompted us to perform a proteomic analysis of antibiotic-induced OMVs.

### Proteogenomic characterization of *A. baumannii* OMVs

**Fig. 3:**
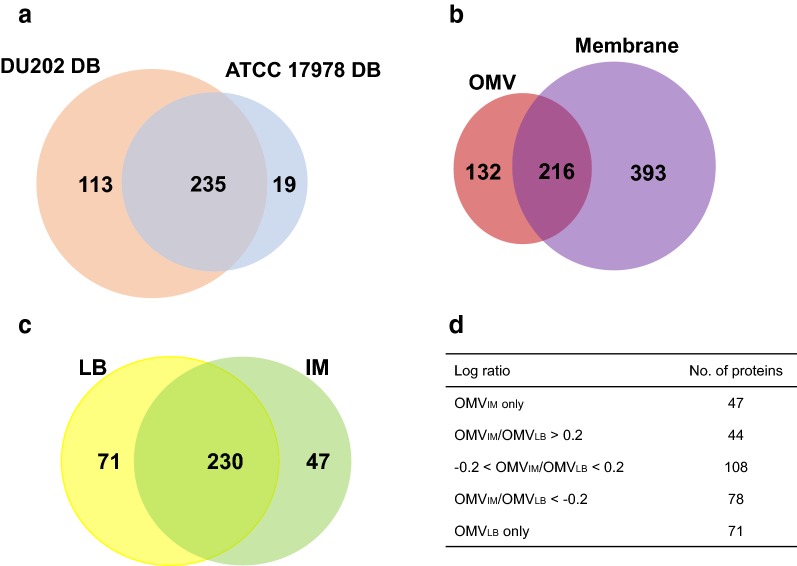
Venn diagrams for comparative proteome analysis. **a** OMV proteomic results based on two indicated genomic databases. **b** Summary of proteomic analysis of two sub-proteomes (OMV vs. bacterial membrane fraction). **c** Comparative proteome analysis of OMV_LB_ and OMV_IM_. **d** Quantitative summary of OMV proteomics. The numbers indicate the identified protein number

In our previous proteomic studies, we used the *A. baumannii* ATCC 17978 genome as a reference genome [15], but in the present work, we updated the reference genome with that of *A. baumannii* DU202. To identify protein components of *A. baumannii* DU202 OMVs, purified OMVs were fractionated by 12% SDS–PAGE and subjected to in-gel tryptic digestion for LC-MS/MS analysis. When using the *A. baumannii* ATCC 17978 genome as a reference, we identified 254 proteins in *A. baumannii* DU202 OMVs (Fig. 3a). A further 113 proteins were identified using the *A. baumannii* DU202 genome, and 19 proteins obtained using the *A. baumannii* ATCC 17978 genome were deleted (Fig. 3a).

Comparative proteomic analysis of purified OMVs and bacterial membrane-associated protein fractions was performed, and as expected, not all protein components of membrane-associated protein fractions were detected in purified OMVs. Indeed, only 35.5% of the protein components (216 of 609 proteins) in membrane-associated protein fractions were also detected in the OMV proteome (Fig. 3b). Spearman correlation analysis values of commonly induced proteins in OMVs and the membrane-associated protein fractions were only 0.45–0.52, indicating a relatively poor correlation between the two proteome datasets (Additional file 1: Figure S1). These results suggest that the protein components of OMVs were differentially enriched and selectively sorted during the segregation of OMVs from host bacteria.

**Fig. 4:**
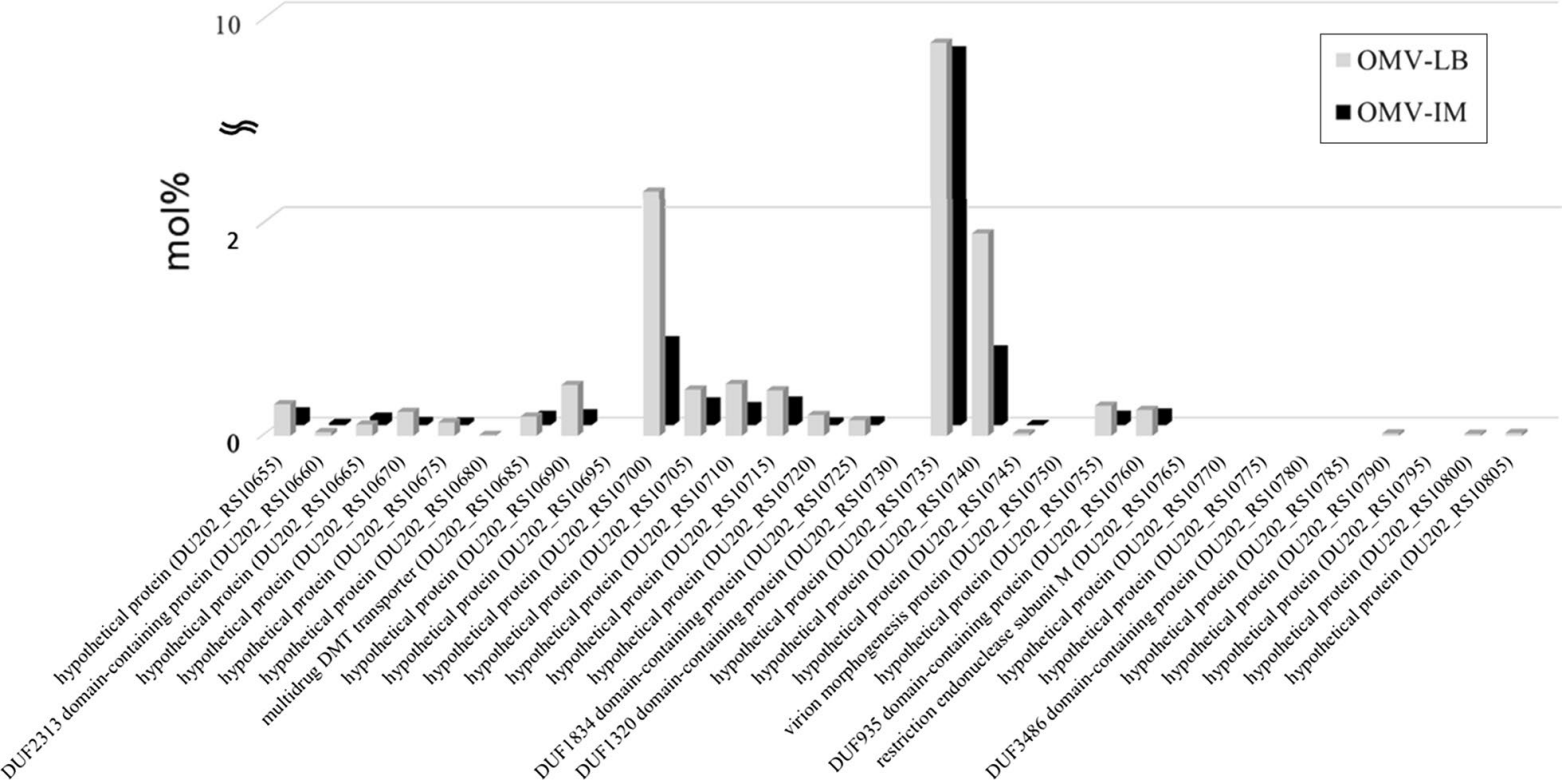
Mu-like prophage gene cluster in *A. baumannii* DU202 and their expression in OMVs

Another interesting result of proteogenomic analysis was the detection of prophage gene clusters in the genome and their expression in OMVs as major protein components (Fig. 4). The PHAST program identified eight gene clusters, including five intact bacteriophage genes, scattered throughout the genome of *A. baumannii* DU202. Proteomic analysis of *A. baumannii* DU202 OMVs revealed that, among these, four bacteriophage gene clusters (three intact and one questionable) were active in the expression of the phage components (Additional file 2: Figure S2). Mu-like prophage major head subunit (DU202_RS10735), phage major capsid proteins (DU202_RS09385) and putative proteins (DU202_RS14035, DU202_RS10700 and DU202_RS14845) were identified as major proteins in purified OMVs (Fig. 4 and Additional file 3: Table S1). Because bacteriophages and OMVs are of a similar size (50–200 nm), we cannot completely exclude the possibility of co-purification of the two particles. Indeed, several studies reported that OMVs form complexes with phages to prevent phage attack [27–29]. However, EM image analysis confirmed the high purity of OMVs (Fig. 1a), suggesting that OMV particles may contain phage proteins as major protein components. Recent genome sequencing of clinical *A. baumannii* strains revealed the presence of phage islands that have been classified as cryptic prophages [30]. Therefore, it was necessary to confirm whether phage proteins induced in clinical *A. baumannii* strains were incorporated into OMVs. Our proteomic results clearly showed differential expression of phage proteins correlated with antibiotic treatment, and about 40% of phage protein expression was downregulated following imipenem treatment (Fig. 4).

Finally, genomic analysis of *A. baumannii* DU202 revealed the presence of four β-lactamase genes in the genome, and the proteomic results demonstrated upregulation of β-lactamase OXA-23 (DU202_RS06415) following exposure to imipenem. In particular, OXA-23 accounted for about 36% of total proteins in OMV_IM_, and was upregulated 9.23-fold compared with OMV_LB_ (Table 1).

**Table 1:** Differential induction of major outer membrane proteins of *Acinetobacter baumannii* DU202 OMV according to imipenem treatment

Locus_tag	Description	Localization	Log ratio^a^	OMV_LB_^b^	OMV_imipenem_^b^
DU202_RS06415	Carbapenem-hydrolyzing class D beta-lactamase OXA-23	Cytoplasmic	0.923	4.3782	36.682
DU202_RS02680	Tail-specific protease	OuterMembrane	0.855	0.037	0.262
DU202_RS15465	Lipoprotein NlpD	Periplasmic	0.69	0.051	0.251
DU202_RS16100	Tol–Pal system beta propeller repeat protein TolB	OuterMembrane	0.53	0.168	0.57
DU202_RS19805	Transporter	OuterMembrane	0.461	0.078	0.225
DU202_RS12145	Outer membrane protein assembly factor BamA	OuterMembrane	0.46	0.111	0.319
DU202_RS11840	TonB-dependent siderophore receptor	OuterMembrane	0.439	0.045	0.123
DU202_RS15930	Putative serine protease	OuterMembrane	0.413	0.326	0.844
DU202_RS18760	Superoxide dismutase (Cu–Zn)	Periplasmic	0.366	0.762	1.77
DU202_RS20255	M23 family peptidase	Periplasmic	0.365	0.051	0.119
DU202_RS01660	Outer membrane protein W precursor	Periplasmic	0.265	0.49	0.903
DU202_RS04315	Outer membrane protein assembly factor BamD	OuterMembrane	0.244	0.106	0.186
DU202_RS17430	Outer membrane protein A precursor	OuterMembrane	0.196	1.775	2.789
DU202_RS00390	FKBP-type peptidyl-prolyl cis–trans isomerase	OuterMembrane	0.178	0.083	0.125
DU202_RS10675	Putative bacteriophage Mu Gp45 protein	Periplasmic	− 0.579	0.112	0.03
DU202_RS16695	Succinate dehydrogenase flavoprotein subunit	Cytoplasmic	− 0.586	0.158	0.041
DU202_RS17820	Preprotein translocase subunit YajC	InnerMembrane	− 0.592	0.18	0.046
DU202_RS14050	Phage head–tail adapter protein	Cytoplasmic	− 0.61	0.149	0.037
DU202_RS09385	Phage major capsid protein	Periplasmic	− 0.632	1.084	0.253
DU202_RS04220	Peptidoglycan-binding protein LysM	Periplasmic	− 0.7	0.12	0.024
DU202_RS05710	Copper resistance protein NlpE	Extracellular	− 0.743	0.447	0.081
DU202_RS09380	HK97 family phage prohead protease	Cytoplasmic	− 0.75	0.214	0.038
DU202_RS10720	Phage tail sheath-like protein	Periplasmic	− 0.767	0.175	0.03
DU202_RS13995	Lytic transglycosylase domain-containing protein	OuterMembrane	− 0.8	0.16	0.025
DU202_RS14000	Methyl-coenzyme M reductase	OuterMembrane	− 0.967	0.482	0.052
DU202_RS16690	Succinate dehydrogenase iron-sulfur subunit	Cytoplasmic	− 1.307	0.154	0.008
DU202_RS09375	Phage portal protein	OuterMembrane	− 1.522	0.718	0.022

^a^Induction ratio was calculated as OMV_LB_ per OMV_IM_

^b^Abundance was indicated as mol%

### Imipenem induces differential expression of surface proteins in *A. baumannii* OMVs

Next, we compared protein contents of OMV_LB_ and OMV_IM_. Of total 348 proteins, OMV_LB_ and OMV_IM_ shared 230 proteins, and 71 and 47 proteins were exclusively expressed in OMV_LB_ and OMV_IM_, respectively (Fig. 3c, d). Above, we showed that OMV_IM_ are more cytotoxic than OMV_LB_, and their protein contents are different from each other. To investigate proteins that may contribute to the cytotoxic activity of OMV_IM_, we focused on differentially expressed proteins between OMV_LB_ and OMV_IM_, especially localized in outer membrane, periplasm and extracellular region. Eight proteases were identified in the proteome of OMVs, all of which were upregulated in the imipenem culture (Additional file 3: Table S1). Of these, putative serine protease (DU202_RS15930), M23 family peptidase (DU202_RS20255) and tail-specific protease (DU202_RS02680) were particularly highly upregulated and predicted as major outer membrane proteins (Table 1). Although the biological functions of these proteases are not yet clear, periplasmic and serine proteases have been linked to pathogenic activities in several pathogenic Gram-negative and Gram-positive bacteria including *P. aeruginosa*, *E. coli* and *Streptococcus pyogenes* [31–34]. Sequence homology analysis showed that putative serine protease (DU202_RS15930) shares significant homology (86–91% coverage, 33–34% identity) with HtrA protease and DegP from various pathogenic bacteria [35, 36]. Putative peptidase S41 (DU202_RS01365) shares high sequence similarity with CtpA of *P. aeruginosa* (76% coverage, 33% identity), which is cytotoxic toward host cells and essential for the type 3 secretion system [31].

Outer membrane proteins and porins (DU202_RS17430, DU202_RS01660, DU202_RS12145, DU202_RS04315 and DU202_RS16100) were also upregulated in OMVs following exposure to imipenem (Table 1). Outer membrane protein A (OmpA, DU202_RS17430) of *A. baumannii* is cytotoxic and involved in biofilm formation as well as adhesion, invasion and apoptosis of host cells [37–39]. In fact, OmpA is shown to contribute in the antimicrobial resistance. Disruption of OmpA gene results in decreased antibiotic resistance of *A. baumannii* [40]. OmpW (DU202_RS01660) is a highly immunogenic protein that elicits protective immunity against *A. baumannii* infections [41]. β-barrel assembly machine (BAM) proteins are outer membrane complexes responsible for folding and insertion of β-barrel outer membrane proteins, and are considered to be strong vaccine candidates in Gram-negative bacteria [42, 43]. In this study, BamA (DU202_RS12145) and BamD (DU202_RS04315) were upregulated by imipenem (Table 1), as was TolB (DU202_RS16100), which increases OMV formation in *Helicobacter pylori* [44].

Peptidyl-prolyl cis–trans isomerases (PPIs) catalyse the cis/trans isomerisation of peptide bonds preceding prolyl residues during protein folding [45]. PPIs have been identified as virulence-associated proteins in bacteria such as *Legionella pneumophila*, *Enterobacteriaceae* and *Yersinia pseudotuberculosis* [46]. Expression of *A. baumannii* DU202 PPI (DU202_RS00390) was upregulated > 1.8-fold in OMV_IM_, and superoxide dismutase (DU202_RS18760) and lipoprotein NlpD (DU202_RS15465) were also induced in OMVs by imipenem (Table 1). These proteins have been linked to virulence in the pathogenic bacteria *Neisseria meningitidis*, *Brucella abortus* and *Yersinia pestis* [47, 48].

### Immunogenic proteins in *A. baumannii* OMV_IM_

**Fig. 5:**
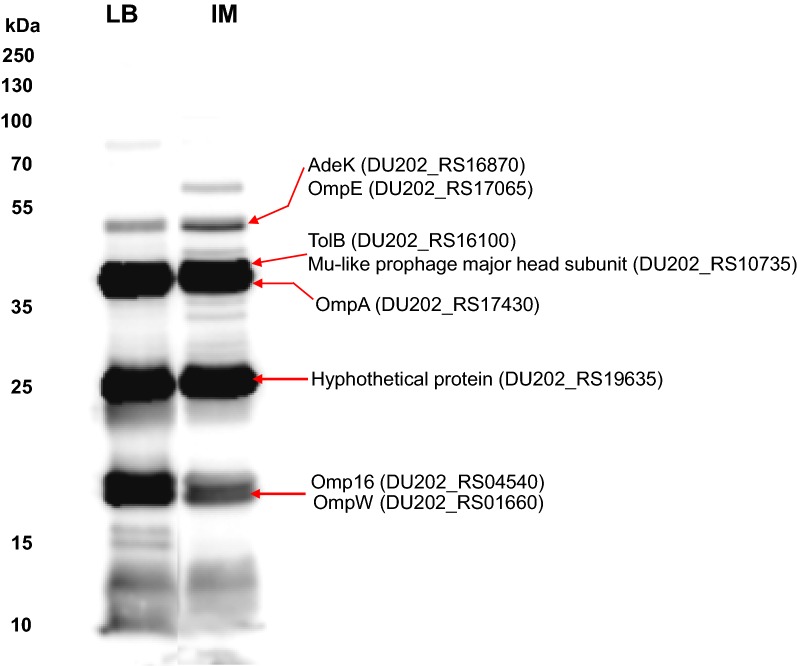
Identification of immunogenic proteins of *A. baumannii* DU202 OMVs. Western blot using *A. baumannii* OMV antiserum revealed major immunogenic proteins. The protein bands were excised from gels identified by tandem mass spectrometry (MS/MS) analysis

To identify proteins with high immunogenic activity among the *A. baumannii* DU202 OMV proteins that may be candidates for diagnostic markers or vaccines, western blotting was performed using the *A. baumannii* DU202 OMV antiserum. In previous studies, OmpA, OmpO and OmpW were identified [6]. Among the identified 348 OMV proteins, eight proteins (AdeK, OmpE, OmpA, TolB, OmpW, lipoprotein Omp16, Mu-like prophage head subunit and hypothetical protein) were predicted to be highly immunogenic (Fig. 5 and Additional file 4: Table S2). Interestingly, all are cell surface proteins (outer membrane or periplasmic) according to the subcellular prediction program, but it was not possible to differentiate between OMV_LB_ and OMV_IM_.

## Conclusions

Treatment of the *A. baumannii* clinical strain DU202 with imipenem increased OMV production, modified OMV proteome components and enhanced pathogenicity toward cultured host cells. *A. baumannii* DU202 includes several prophage gene clusters in its genome, some of which are highly expressed in OMVs. Our proteogenomic analysis successfully identified several unique genomic characteristics of OMVs from a clinical *A. baumannii* strain that could prove useful for developing antibiotic agents in the future.

## Additional files

**Additional file 1: Figure S1.** Analysis of spearman correlation of commonly induced proteins of the OMVs and the membrane-associated protein fraction.

**Additional file 2: Figure S2.** Expression of phage genes in *A. baumannii* DU202 OMV. **a** Complete genome of *A. baumannii* DU202 and proteins expression in OMVs. **b** Protein expression pattern of bacteriophage gene clusters in OMVs.

**Additional file 3: Table S1.** Comparative proteomic analysis of *A. baumannii* DU202 OMVs.

**Additional file 4: Table S2.** Proteomic analysis of immunogenic proteins of *A. baumannii* DU202 OMVs.

## Abbreviations

OMVs: outer membrane vesicles
1DE-LC-MS/MS: one-dimensional electrophoresis and liquid chromatography combined with tandem mass spectrometry
BAM: β-barrel assembly machine
PPIs: peptidyl-prolyl cis–trans isomerases
MDR: multidrug-resistant
Bap: biofilm-associated protein
LB: Luria–Bertani
PBS: phosphate-buffered saline
TEM: transmission electron microscopy
SDS–PAGE: sodium dodecyl sulphate–polyacrylamide gel electrophoresis
TFA: trifluoroacetic acid
LC-MS/MS: liquid chromatography combined with tandem mass spectrometry
MS/MS: tandem mass spectrometry
emPAI: the exponentially modified protein abundance index
FBS: fetal bovine serum
FITC: fluorescein isothiocyanate
PI: propidium iodide
TBS: TRIS-buffered saline
