# Supplementary figures and images for: Antibiotic treatment modulates protein components of cytotoxic outer membrane vesicles of multidrug-resistant clinical strain, Acinetobacter baumannii DU202
Source: Clin Proteomics. 2018 Aug 31;15:28. doi: 10.1186/s12014-018-9204-2 (PMC6118003; doi:10.1186/s12014-018-9204-2)

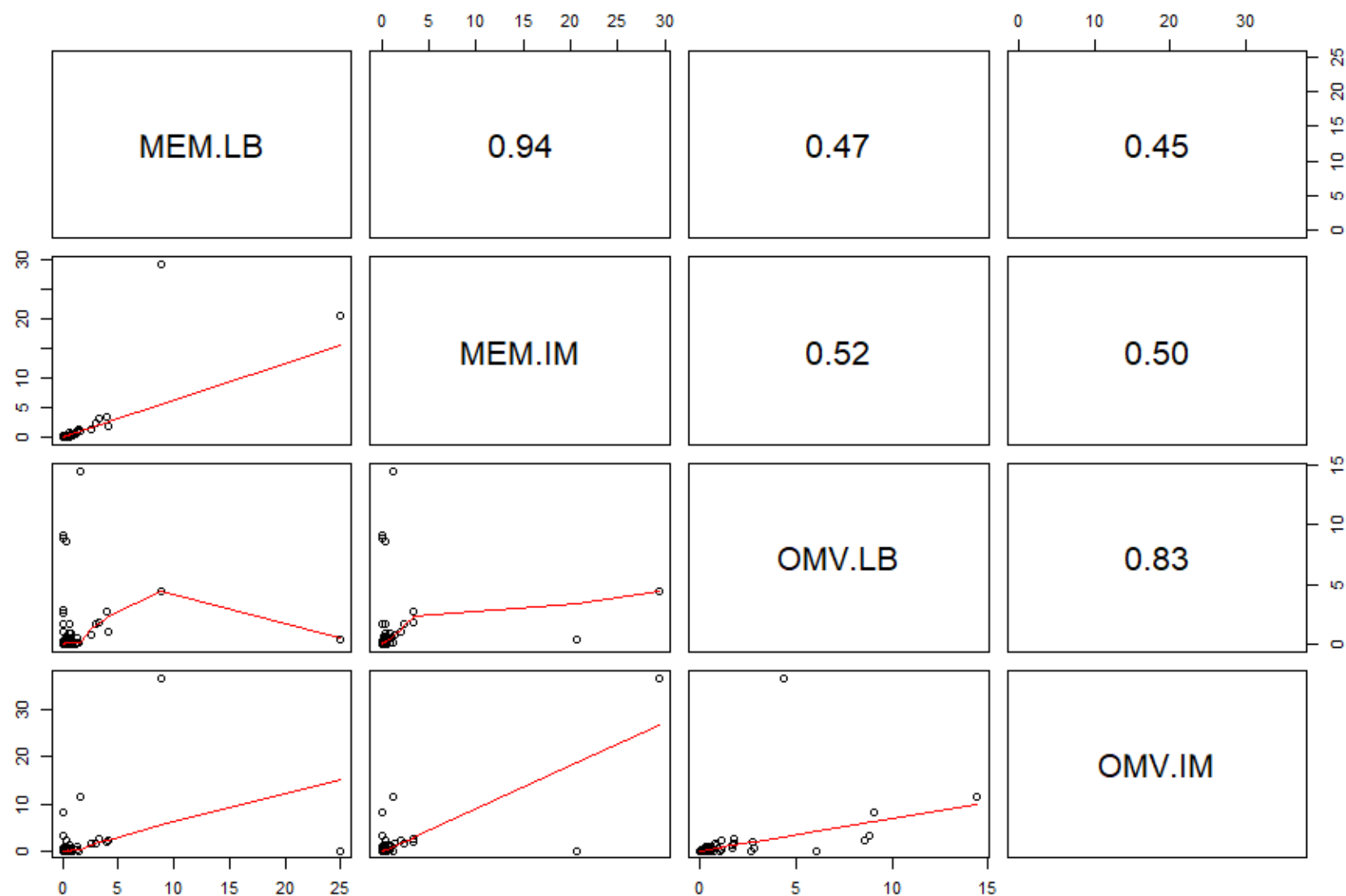

Supplement: Supplementary file 1 — Additional file 1: Figure S1. Analysis of spearman correlation of commonly induced proteins of the OMVs and the membrane-associated protein fraction. [file 12014_2018_9204_MOESM1_ESM.pdf]
