# Supplementary material for: Antibiotic treatment modulates protein components of cytotoxic outer membrane vesicles of multidrug-resistant clinical strain, Acinetobacter baumannii DU202
Source: Clin Proteomics. 2018 Aug 31;15:28. doi: 10.1186/s12014-018-9204-2 (PMC6118003; doi:10.1186/s12014-018-9204-2)
Supplement: Supplementary file 2 — Additional file 2: Figure S2. Expression of phage genes in A. baumannii DU202 OMV. a Complete genome of A. baumannii DU202 and proteins expression in OMVs. b Protein expression pattern of bacteriophage gene clusters in OMVs. [file 12014_2018_9204_MOESM2_ESM.pptx]

## Slide 1
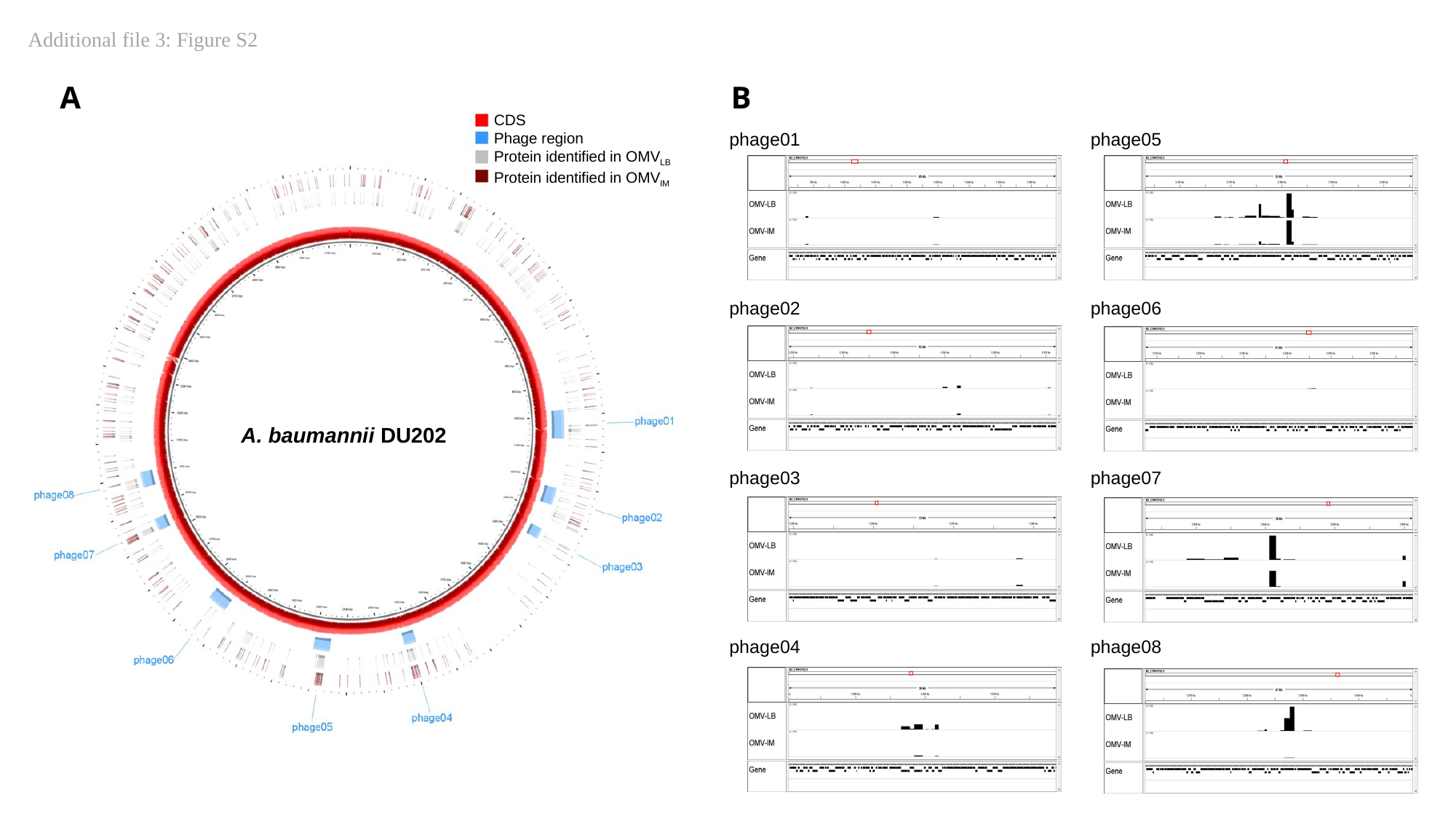

Additional file 3: Figure S2
A
B
CDS
Phage region
Protein identified in OMVLB
Protein identified in OMVIM
A. baumannii DU202
phage01
phage05
phage06
phage02
phage07
phage03
phage08
phage04
