# Supplementary material for: Antibiotic treatment modulates protein components of cytotoxic outer membrane vesicles of multidrug-resistant clinical strain, Acinetobacter baumannii DU202
Source: Clin Proteomics. 2018 Aug 31;15:28. doi: 10.1186/s12014-018-9204-2 (PMC6118003; doi:10.1186/s12014-018-9204-2)
Supplement: Supplementary file 4 — Additional file 4: Table S2. Proteomic analysis of immunogenic proteins of A. baumannii DU202 OMVs. [file 12014_2018_9204_MOESM4_ESM.docx]

**Table S2. Proteomic analysis of immunogenic proteins of *A. baumannii* DU202 OMV**

| **Locus_tag** | **Description Name** | **Mw** | **pI** | **Length** | **Score** |
| --- | --- | --- | --- | --- | --- |
| DU202_RS16870 | RND  efflux system, AdeK | 52770 | 9.02 | 484 | 62 |
| DU202_RS17065 | Outer  membrane protein E | 53090 | 5.15 | 503 | 52 |
| DU202_RS16100 | Translocation protein TolB | 46544 | 9.19 | 426 | 106 |
| DU202_RS10735 | Mu-like prophage major head subunit | 34269 | 6.86 | 310 | 51 |
| DU202_RS17430 | Outer membrane protein A precursor | 38427 | 5.32 | 356 | 126 |
| DU202_RS19635 | Hypothetical protein | 25388 | 4.49 | 230 | 127 |
| DU202_RS04540 | Outer  membrane lipoprotein omp16 precursor | 22472 | 9.3 | 217 | 142 |
| DU202_RS01660 | Outer membrane protein W precursor | 21213 | 5.56 | 193 | 163 |
